# Thermo-Reversible Hybrid Gels Formed from the Combination of Isotactic Polystyrene and [Fe(II) (4-Octadecyl-1,2,4-Triazole)_3_(ClO_4_)_2_]_n_ Metallo-Organic Polymer: Thermal and Viscoelastic Properties

**DOI:** 10.3390/polym11060957

**Published:** 2019-06-01

**Authors:** Coro Echeverría, Miguel Rubio, Daniel López

**Affiliations:** Instituto de Ciencia y Tecnología de Polímeros (ICTP-CSIC), C/Juan de la Cierva 3, 28006 Madrid, Spain; sambaya.music@gmail.com

**Keywords:** metallo-organic polymer, isotactic polystyrene, hybrid gel, thermo-reversible gel

## Abstract

Nano-sized one-dimensional metallo-organic polymers, characterized by the phenomenon of spin transition, are excellent candidates for advanced technological applications such as optical sensors, storage, and information processing devices. However, the main drawback of this type of polymers is their fragile mechanical properties, which hinders its processing and handling, and makes their practical use unfeasible. To overcome this problem, in this work, hybrid thermo-reversible gels are synthesized by combination of a metallo-organic polymer and isotactic polystyrene (iPS) in cis-decaline. A detailed investigation of the thermal and viscoelastic properties of the hybrid gels, in terms of iPS and metallo-organic polymer concentration is performed by means of differential scanning calorimetry and oscillatory rheology, respectively. From the analysis of the thermal properties, three transitions have been determined upon heating: Monotectic transition of the iPS gel, melting of the iPS gel, and melting of the metal-organic polymer gel, which suggest that the gels of the two polymers are formed independently in the hybrid gel, as long as the two polymers are in concentrations above the corresponding critical gelation concentrations. Results regarding viscoelastic properties and morphology confirmed that hybrid gels consisted of an interpenetrated network of polymer gels, formed by iPS and metallo-organic poymer gels growing independently.

## 1. Introduction

Nano-sized one-dimensional metallo-organic polymers have demonstrated to be excellent candidates for advanced technological applications such as optical sensors, storage, and information processing devices, etc. [1,2,3,4,5,6]. In particular, polymers of the transition metal ions family with d4–d7 electronic configuration and octahedral symmetry constitute a group of new functional materials with very interesting optical, magnetic, and electronic properties [1,2]. These polymers are characterized by the phenomenon of spin transition, as the polymer can present two electronic states (corresponding to two magnetic states) switchable by the effect of temperature, pressure, light, etc. [6,7]. The change from one state to another is usually accompanied by changes in the physical properties of the material, such as changes in color, dielectric constant, etc., which confer additional applications to these materials [8].

However, these polymers present serious problems such as fragility from the mechanical point of view, instability to oxidation, the difficulty of processing and handling, which make their practical use unfeasible. Therefore, the main challenge of these systems for the development of their potential applications is how to transfer the inherent properties of the polymer in solid state to systems suitable for technological applications. For certain applications, a specific spatial arrangement is crucial. Research performed over the last decades proposed the preparation of SCO materials as thin-films [9,10], liquid crystals [11], and as supramolecular gels [12,13]. In order to overcome this problem, in our previous study, electrospun fibers of blends of the [Fe(II) (4-octadecyl-1,2,4-triazole)_3_(ClO_4_)_2_]_n_ metallo-organic polymer and atactic polystyrene were developed. [14] This approach allowed to obtain fibers showing the mechanical properties of the organic polymer (atatic polystyrene) but retaining the magnetic properties of the pure metallo-organic polymer [14]. Prior to that work, we reported the development of [Fe(II) (4-octadecyl-1,2,4-triazole)_3_(ClO_4_)_2_]_n_ metallo-organic polymer gel and the effect of the solvent on the gelation properties [15]. We demonstrated, by means of Small Angle Neutron Scattering (SANS) and rheology, that the responsibility for the gelation is with the side-by-side aggregation into fibers of the individual metallo-organic polymer chains [13]. The formation of hybrid thermo-reversible gels from organic and metallo-organic polymers is also postulated as an interesting technological alternative. The obtained hybrid materials would fulfil the mechanical requirements due to the presence of the organic polymer, and open a new field of applications with the additional functionalities of the metallo-organic polymer [16]. For the fabrication of this kind of materials, it is necessary to find an appropriate solvent in which the polymers can form gels, or at least one of the polymers form a gel and induce the gelation of the second polymer. For instance, Dasgupta et al. [17] developed a study in which hybrid thermo-reversible gels are formed from organic polymers (atactic, isotactic, and syndiotactic polystyrene) and organogels (prepared from trans-oligo(p-phenylenvynilene) molecules) in organic non-polar solvents: cis-decalin, trans-decalin, and benzene. The obtained hybrid gel is composed of two fiber-like networks, which correspond to each polymer growing independently.

Therefore, in this work, hybrid thermo-reversible gels are synthesized by combination of a metallo-organic polymer and isotactic polystyrene in cis-decaline. In this case, cis-decaline is the solvent in which both polymers form gels. In addition, the viscoelastic properties and the morphology of thermo-reversible hybrid gels are also studied in order to determine the compatibility of the two gels as well as the properties of the resulting system.

## 2. Materials and Methods

**Materials.** For the synthesis of 4-octadecyl-1,2,4-triazol, monoformylhydrazine (Aldrich, St. Quentin Fallavier, France), triethylorthoformate ((Fluka, Seelze, Germany), octadecylamine (Fluka), and 1-pentanol (Aldrich) were used. Monoformylhydrazine and 1-pentanol were dried before use. For the synthesis of the iron complex Iron (II), Perchlorate (Aldrich) and ethanol (Merck, Kenilworth, NJ, USA) were used without previous purification. Isotactic polystyrene (90% isotactic) (iPS) PS2 (Rapra Technology, Akron, OH, USA), an average molecular weight of 136,000 g/mol and a polydispersity index of 3.8, was used. Cis-decalina (Aldrich) was used as solvent for the gel preparation.

The metallo-organic polymer [Fe(II) (4-octadecyl-1,2,4-triazole)_3_(ClO_4_)_2_]_n_ was obtained by a method reported previously [15]. Gels of metallo-organic polymer were obtained by dissolving the polymer in cis-decaline at 100 °C. Then, the obtained solution was cooled down to 0 °C in an iced bath until the gel is formed. In the case of iPS pure gels, the preparation was similar except for the temperature applied. An appropriate iPS content was dissolved in cis-decaline at 180 °C. The homogeneous solutions were then cooled down to 0 °C in an ice bath. In both cases, the cooling process needs to be fast enough to prevent polymer crystallization. Hybrid gels were obtained from the preparation of independent homogeneous solution of both polymers in cis-decaline at 100 °C for the metallo-organic polymer and 180 °C for the solution of iPS. Then, both solutions were mixed and stirred at 100 °C to form a homogeneous mixture that was further cooled down to 0 °C to obtain the gel.

For this study, pure iPS gels with concentrations of polymer in the range of 2% to 9% (m/v) were prepared in cis-decaline. For the hybrid gels, two different sets of samples were prepared in cis-decaline: (i) Hybrid gels with a fixed concentration of iPS (4% (w/v)) and metallo-organic polymer concentration in the range of 0.1% to 6% (m/v); (ii) hybrid gels with a fixed concentration of metallo-organic polymer (1.4% (w/v)) and iPS concentrations in the range of 1% to 7% (m/v).

**Methods.** Thermal characterization of pure iPS and hybrid gels were performed in a Perkin-Elmer DSC 7 Instrument (Whatmann, Clifton, NJ, USA). Heating and cooling experiments were carried out at a rate of 5 °C·min^−1^. The test was conducted as follows: Sample was kept at 80 °C during 1 min, then a cooling sweep test was performed up to −5 °C. This temperature was held for 1 min and the sample was subsequently subjected to a heating sweep test going up to 80 °C. The transitions as well as enthalpies generated along the process were determined from the peaks in the thermograms obtained in the heating and cooling modes, respectively.

Rheological studies were performed using a controlled stress oscillatory rheometer TA Instruments AR1000 (New Castle, England). Rough 20 mm diameter parallel plates were used for all the samples: (i) Torque sweep in a range of 0.1 to 20,000 μN·m test was performed to determine the Linear Viscoelastic Region (LVR) at a constant non-destructive frequency of 1 Hz and at −1 °C; (ii) temperature sweep tests in the range of −1 to 70 °C at a heating rate of 2.5 °C·min^−1^ in the LVR were performed.

For the morphological characterization, topographical images were taken using a multimode atomic force microscopy (AFM) with the control system Nanoscope IIe (Veeco Instruments, Santa Barbara, CA, USA) in the tapping mode. For the sample preparation, gels were first dried at room temperature during 24 h prior to their observation. Although some solvent could still be trapped in the polymer, it did not affect the quality of the measurement and obtained images.

## 3. Results and Discussion

In this work, hybrid gels composed of iPS and [Fe(II) (4-octadecyl-1,2,4-triazole)_3_(ClO_4_)_2_]_n_ metallo-organic polymer were prepared and the effect of each component concentration in the gel formation was analyzed. For this study, two sets of hybrid gels were prepared: (i) Hybrid gels with a fixed iPS concentration (4%, g/mL) and variable metallo-organic polymer concentration and (ii) hybrid gels with fixed metallo-organic polymer concentration (1.4%, g/mL) and variable iPS content. The fixed concentrations of 4% for iPS and 1.4% for metallo-organic polymer were selected, taking into account that this is the critical concentration above which each polymer is capable to form gel independently. In order to understand the formation and the properties of the obtained hybrid gels, we studied the thermal, viscoelastic, and morphological properties of the hybrid gels.

### 3.1. Thermal Properties of Hybrid iPS/Metallo-Organic Polymer Gels

**Figure 1:**
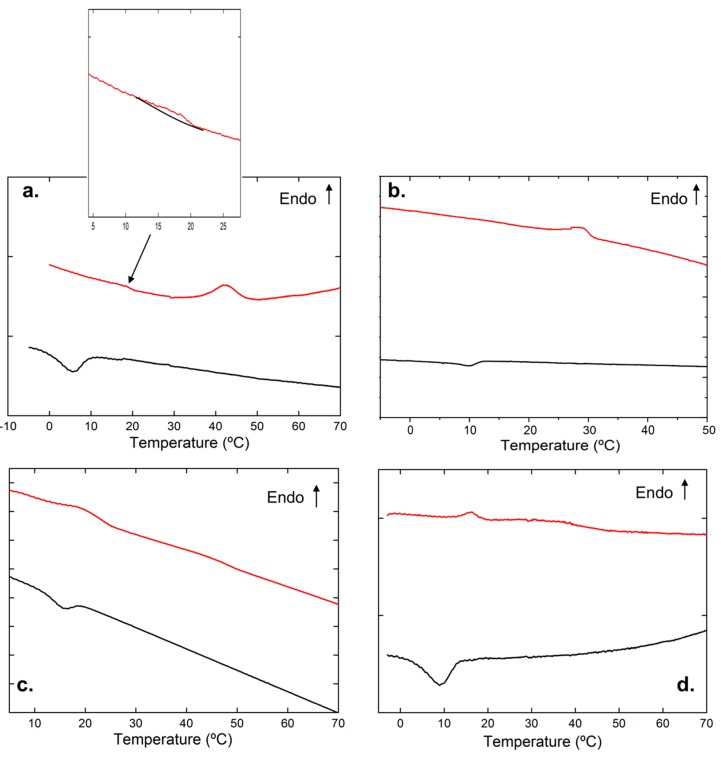
Representative differential scanning calorimetry curves obtained on cooling (red) and on heating (black) for: (**a**) Pure iPS gel in cis-decaline (4%, g/mL) (inset: Amplification of the first endothermic peak at 18 °C), (**b**) metallo-organic polymer gel in cis-decaline (0.9%, g/mL), (**c**) hybrid gel iPS/metallo-organic polymer (iPS = 4%, g/mL and metallo-organic polymer = 5.1%, g/mL), and (**d**) hybrid gel iPS/metallo-organic polymer (metallo-organic polymer = 1.4%, g/mL and iPS = 5.4%, g/mL).

Figure 1a shows the thermogram corresponding to pure iPS gel in cis-decaline (4%, g/mL). As observed from the plot, the iPS gel exhibits two endothermic peaks at 18 and 43 °C upon heating and a single exothermic peak at 6 °C when cooling (See Table 1). The temperature at which this first endothermic peak occurs is independent of the iPS concentration, and corresponds to the monotectic transformation derived from the phase separation effect involved in the gelation phenomena [18,19,20,21]. The second peak corresponds to the iPS gel melting temperature, which varies with iPS concentration. As it is known, the iPS/cis-decalin is a “non-equilibrium” system. In this type of system, the speed at which a polymer solution is cooled down in a given solvent governs the formation of a determined molecular structure, with a greater or lower degree of organization at the molecular level. Rapid sub-cooling leads to the formation of the gel, while sufficiently slow cooling results in the formation of the crystalline state. Between these two extremes, there are two crystalline phases: Phase s and phase p. Except for the crystalline state, the remaining phases contain intercalated solvent molecules. The gel state has an order of nematic type, while the phase s presents characteristics of a smectic arrangement, in which the folding of polymer chains begins to occur. Phase p is less solvated and can be defined as a peritectic system [18,22,23].

The DSC curve of the pure metallo-organic polymer gel (0.9%, g/mL) in cis-decaline is presented in Figure 1b. The thermogram exhibits an exothermic peak at 11 °C when cooling, which is related to gel formation and an endothermic peak of gel melting at 28 °C upon heating.

In Figure 1c,d, thermograms corresponding to iPS /hybrid gels are shown. Figure 1c corresponds to a representative hybrid gel prepared from a fixed iPS concentration (4%, g/mL) with variable concentration of metallo-organic polymer (in this case, 5.1%, g/mL). As observed from the figure, the hybrid gel exhibits an endothermic peak at 20 °C and a wide endotherm where two peaks are vaguely noticeable upon heating (at approximately 30 and 43 °C). The temperature of the first peak, 20 °C, is similar to that observed in pure iPS gel thermogram (Figure 1a); therefore, this peak corresponds to the monotectic transformation of iPS gel. Regarding the two peaks subtly observed in the wide endotherm, the peak appearing at 30 °C coincides with that observed in Figure 1b corresponding to the melting of the metallo-organic polymer. Similar results are observed in Figure 1d, where the thermograms corresponding to an example of hybrid gel prepared from a fixed metallo-organic polymer concentration (1.4%, g/mL) and variable content of iPS (5.4%, g/mL, for this representative graph) are shown. Three endothermic peaks are depicted in the graph upon heating: 16, 28, and 38 °C (See Table 1), which may correspond to the monotectic transformation of iPS gel, the melting temperature of the metallo-organic polymer gel, and melting temperature of the iPS gel, respectively.

**Figure 2:**
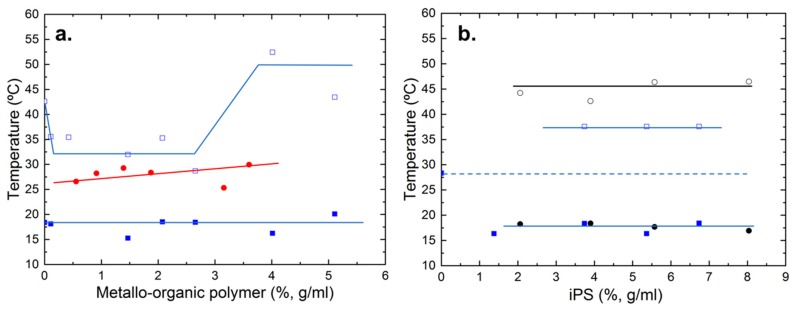
Partial phase diagrams of iPS gels, metallo-organic polymer gels, and hybrid gels obtained upon heating. To build this diagram, first and third endothermic peaks of the hybrid gels corresponding to the iPS gel were chosen: (**a**) Partial phase diagram obtained upon heating for the iPS/metallo-organic polymer/cis-decaline hybrid gels with a fixed concentration of iPS (4%, g/mL) and variable metallo-organic polymer gel. Pure metallo-organic polymer gel (red circle), first (blue square), and third endothermic peak (blue empty square) of the hybrid gel are plotted. (**b**) Partial phase diagram obtained on heating for the iPS/metallo-organic polymer/cis-decaline hybrid gels with a fixed concentration of metallo-organic polymer (1.4%, g/mL) and variable concentration of iPS: First (black circle, full symbol) and third endothermic peak of pure iPS (black circle, empty symbol), and first (blue square, full symbol) and third endothermic peak of the hybrid gel (blue square, empty symbol) are represented.

In order to better understand the effect of the composition in the formation of the hybrid gel, in Figure 2a,b, partial phase diagrams are shown. In particular, in Figure 2a, the partial phase diagram of the hybrid gel with a fixed iPS concentration (4%, g/mL) and the pure metallo-organic polymer gel are shown, both as a function of metallo-organic polymer content. As it can be seen, the melting temperature of the pure metallo-organic polymer gel increases slightly with concentration. As previously studied, this is due to an increase in the crosslink density as the concentration of the polymer increases [15,24]. Additionally, it was determined that the physical gel formation was induced by the crystallization of the metallo-organic polymer chains. For the hybrid gel (with a fixed iPS content of 4 g/mL), two different sets of points are represented, each set corresponding to the first and third endothermic peak observed in the thermograms (Figure 1c). The temperature at which the first peak appears remains constant with the increase of the metallo-organic polymer content, indicating that this first peak corresponds to the monotectic transformation of iPS gel.

The temperature of the third endothermic peak corresponding to the hybrid gel decreases with respect to the peak related to the pure iPS gel at low metallo-organic polymer concentrations. This result is related to the fact that the metallo-organic polymer is not capable of forming a gel on its own at low concentrations, besides hindering the gelation of iPS. Then, the melting temperature stabilizes for intermediate concentrations of metallo-organic polymer (up to 2.6%, g/mL). At such concentrations, the metallo-organic polymer is capable of forming a gel, however the melting temperatures of the iPS in the hybrid gel are still lower than those of the pure iPS gel. Although the hybrid gel is formed, the metallo-organic polymer forms a “weak” gel at this intermediate concentration, and, therefore, iPS dilution phenomena continue to occur.

When the metallo-organic polymer concentration is high enough to form a gel, and the gelation of the iPS is not hampered, the melting temperature of the iPS in the hybrid gel is higher than the melting temperature of the pure iPS gel. At this point, phase separation arises and separate gelation of the two systems occur.

Similar analysis is performed for the results collected in Figure 2b. In this case, partial phase diagrams as a function of iPS content are shown for pure iPS polymer gel and for hybrid gels with a fixed metallo-organic polymer concentration (1.4%, g/mL). In the case of pure iPS gel, it is observed that both the temperature where the monotectic transition peak appears and the temperature at which the melting peak occurs remain constant with iPS concentration. Regarding the hybrid gel, two series of points corresponding to the first and third peak of the thermograms (Figure 2b) are plotted. From the graph, it is observed that: i) The temperature of the first peak remains constant with the concentration of iPS and ii) the third peak, which also remains constant, only appears above iPS concentrations of 3.5% (g/mL).

As mentioned before, the third peak observed upon heating in the thermograms corresponds to the melting of the iPS gel. The fact that melting temperature of the hybrid gel occurs at temperatures lower than those of iPS gel responds to a dilution phenomenon derived from the presence of the metal-organic polymer in the mixture. However, the temperature at which this peak appears hardly varies with the concentration of iPS, which could indicate the slight influence of the metallo-organic polymer on the gelation of the hybrid system.

Therefore, the obtained results indicate that the hybrid gels are formed by the independent gelation of both systems: iPS/cis-decalin and metallo-organic polymer/cis-decalin. Thus, the limiting parameter for the formation of the hybrid gel is the critical concentration of each polymer independently. Therefore, the hybrid gel will be formed as long as the two polymers are in concentrations above the critical gelation concentrations, which increase in relation to the pure components.

### 3.2. Viscoelastic Properties of the Hybrid iPS/Metallo-Organic Polymer Gels—Effect of iPS and Metallo-Organic Polymer Concentrations

**Figure 3:**
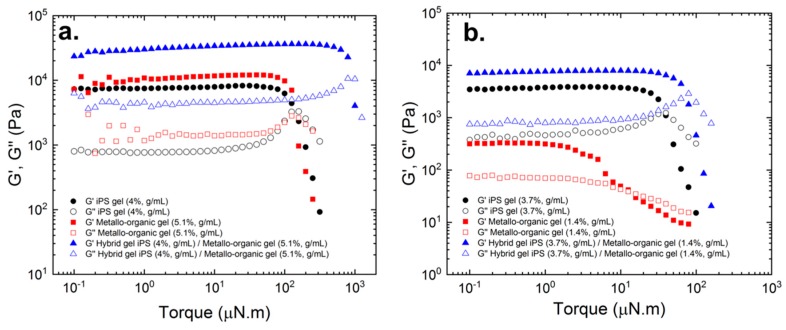
Storage (full symbols) and loss (empty symbols) modulus as a function of oscillation torque, at a constant frequency of 1 Hz and a temperature of −1 °C for: (**a**) Pure iPS gel, 4%, g/mL (black), pure metallo-organic polymer gel 5.1%, g/mL (red), and representative hybrid gel (blue) with a fixed concentration of iPS (4%, g/mL) and variable concentration of metallo-organic polymer (5.1%, g/mL). (**b**) Pure iPS gel, 3.7% g/mL (black), pure metallo-organic polymer gel, 1.4%, g/mL (red), and representative hybrid gel (blue) with a fixed concentration of metallo-organic polymer (1.4%, g/mL) and variable concentration of iPS (3.7%, g/mL).

In Figure 3a,b, the evolution of storage G’ and loss modulus G’’ with the oscillation torque is described for pure iPS gels, pure metallo-organic polymer gels, and for hybrid iPS/metallo-organic polymer gels. The three gels describe a common rheological comportment showing G’ higher than G’’, which is indicative of a solid-like behavior. G’ and G’’ show a plateau up to a critical torque value where both moduli decrease abruptly and crossover modulus (G’ = G’’). The decreasing and crossover of both moduli entails the transition from a solid-like to liquid-like behavior. From these graphs, we could also determine the linear viscoelastic regime (LVR) where the values of G’ and G’’ are independent of the applied torque [16]. As observed from Figure 3a,b, the values of the storage modulus and the critical torque of the hybrid gels are greater than those of the pure polymers separately. This suggests the interaction or interpenetration of the two networks.

**Figure 4:**
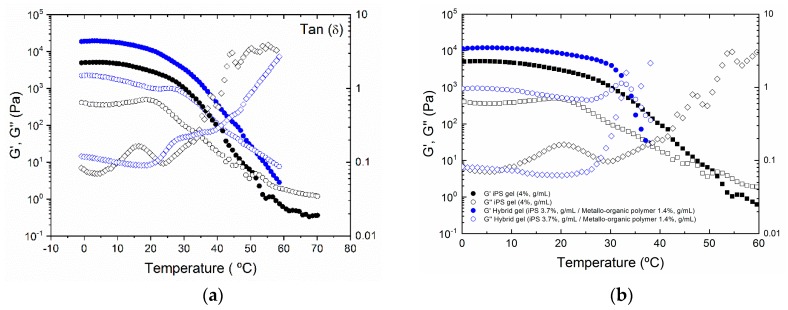
Storage (full symbols) and loss (empty symbols) modulus as a function of oscillation torque measured for: (**a**) Pure iPS gel, 4%, g/mL (black), hybrid gel (blue) with a fixed concentration of iPS (4%, g/mL) and variable concentration of metallo-organic polymer (5.1%, g/mL) and (**b**) pure iPS gel, 4%, g/mL (black), and representative hybrid gel (blue) with a fixed concentration of metallo-organic polymer (1.4%, g/mL) and variable concentration of iPS (4%, g/mL).

In Figure 4a,b, the evolution of storage and loss modulus with temperature for the pure iPS gel and hybrid iPS/metallo-organic polymer gels is represented. If we focus on the temperature dependence of iPS (Figure 4a,b), two pseudo-equilibrium plateaus can be observed, where G’ is independent of the temperature. Between the two plateaus there is a transition, detected by the presence of a maximum in the loss tangent (tan δ) at 18 °C. At higher temperatures, the drop of the modulus and subsequent crossover occurs, which marks the melting of the gel (53 °C). The first transition could correspond to the monotectic transition of the iPS gel, also observed in the thermogram of the pure iPS gel (Figure 1a). The temperature at which this transition occurs is different depending on the experimental technique used to determine it. Temperatures determined by rheometry and DSC do not coincide, since the speeds at which the heating scans have been carried out are not the same. In DSC, a speed of 5 °C/min has been used, while in rheometry, the speed has been 2.5 °C/min. From the analysis performed on the hybrid gel with a fixed concentration of iPS (4%, g/mL) and variable concentration of metallo-organic polymer (5.1%, g/mL), two plateaus defining two pseudo-equilibrium storage moduli are also observed. In this case, this first transition between both plateaus, also determined by a maximum of the loss tangent, occurs at 25 °C. The crossover of the modulus determining the melting of the hybrid gel occurs at the same temperature as pure iPS gel, 52 °C. Similar conclusions are obtained when comparing pure iPS (4%, g/mL) with the hybrid gel with a fixed concentration of metallo-organic polymer (1.4%, g/mL) and variable concentration of iPS (3.7%, g/mL) (Figure 4b). A difference is only found in the temperature at which the melting takes place: In the hybrid gel, melting occurs at 33 °C, being significantly lower than the melting temperature of the pure iPS gel (53 °C).

**Figure 5:**
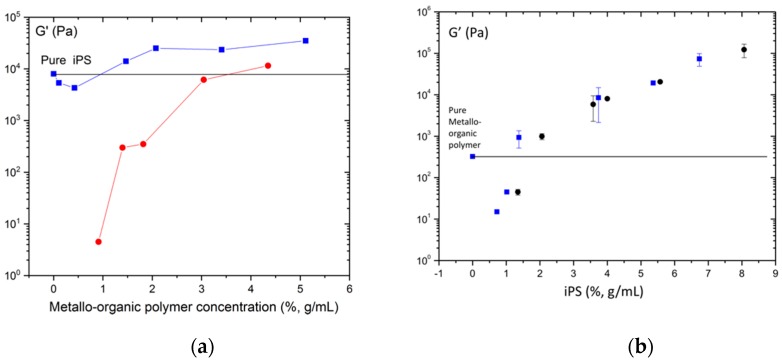
(**a**) Equilibrium storages modulus plateau as a function of metallo-organic polymer concentration for hybrid gels with a fixed iPS concentration of 4%, g/mL (blue square) and for pure metallo-organic polymer gels (red circle); (**b**) equilibrium storages modulus plateau as a function of iPS concentration for hybrid gels with a fixed metallo-organic polymer concentration of 1.4%, g/mL (blue squares) and for pure iPS gels (black circle).

In Figure 5a, we have represented the equilibrium storage modulus G’ as a function of metallo-organic polymer concentration for hybrid gels with fixed iPS concentration (4%, g/mL) and for pure metallo-organic polymer gels. As it can be observed, the equilibrium storage modulus of pure metal-organic polymer gels increase with concentration; besides, the value of storage modulus appears to be above the equilibrium storage modulus of the pure iPS gel of 4% (g/mL) at a concentration of metal-organic polymer close to 3.6% (g/mL). In the case of the hybrid gel (with fixed iPS concentration), the equilibrium storage modulus decreases up to a concentration of 0.5% (g/mL), from which it begins to increase, ending at a value that is higher than the one shown by pure iPS gel (4%, g/mL) above a metallo-organic polymer concentrations of 1% (g/mL). The equilibrium storage modulus of the hybrid gels is always higher than those of the pure metal-organic polymer gels with the same metallo-organic polymer concentration. However, the equilibrium storage modulus of the hybrid gels with metallo-organic polymer concentrations below 1% (g/mL) are lower than the equilibrium value of the pure iPS gel of 4% (g/mL). At such low concentrations (>1%, g/mL), the metallo-organic polymer is not able to form gel on its own, furthermore preventing the gelation of the iPS. This could be explained by a hindrance of the rigidification of the iPS chains or a process of dilution of the iPS in the mixture because of the presence of metallo-organic polymer. When the concentration of metallo-organic polymer in the hybrid gel is enough for the its gelation to take place, then the equilibrium storage modulus increases. This augment could be due to the formation of an interpenetrated network of iPS and metallo-organic polymer gels.

We also analyzed the effect of iPS concentration in the equilibrium storage modulus of hybrid iPS/metallo-organic polymer gel with fixed metallo-organic polymer content (1.4%, g/mL). As observed from Figure 5b, the equilibrium storage modulus of pure iPS gels increases with the concentration of iPS. In the case of the hybrid gels, those with an iPS concentration below 1.2% (g/mL) have lower equilibrium storage modulus values than the pure metal-organic polymer gel. At iPS concentrations below 1.2% (g/mL), the iPS is not capable of gelifying and it could probably inhibit the gelation of the metallo-organic polymer. However, above 1.2% iPS concentration, hybrid gels present equilibrium storage modulus higher than the storage modulus of the pure metallo-organic polymer gel. Moreover, above iPS concentration of 3.5%, g/mL, the equilibrium storage modulus of both pure iPS gel and hybrid gel become similar. This could be due to the fact that the value of the equilibrium storage modulus of the pure metal-organic polymer gel is too low to contribute significantly to the value of the equilibrium modulus of the hybrid gel.

**Figure 6:**
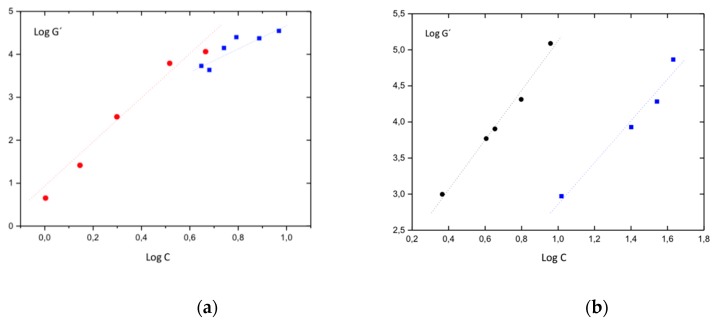
(**a**) Double logarithmic plot of the equilibrium storages modulus plateau as a function of metallo-organic polymer concentration for hybrid gels with a fixed iPS concentration of 4%, g/mL (blue square) and for pure metallo-organic polymer gels (red circle) and (**b**) as a function of iPS concentration for hybrid gels with a fixed metallo-organic polymer concentration of 1.4%, g/mL (blue squares) and for pure iPS gels (black circle).

In Figure 6, the double logarithmic plots of the equilibrium storages modulus as a function of metallo-organic polymer concentration (Figure 6a) and iPS concentration (Figure 6b) are represented for both hybrid gels and pure metallo-organic polymer and iPS gels, respectively. The obtained plots have been analyzed using a theoretical model based on de Gennes scaling law, in which the moduli are related to the concentration as follows: G α C^n^ [25]; n being an exponent that depends on the conformation of the polymer chains linking the connection points.

As it has already been indicated in our previous works, these correlations were developed for chemical gels but they were already applied for physical gels [15]. Since the storage modulus of the studied gels describe two pseudo-equilibrium moduli where G ≠ G(T), it is possible to indicate that the obtained gels are formed by networks presenting enthalpic elasticity. For this type of network, there is an approximation that correlates the equilibrium storage modulus with the concentration through the fractal dimension (υ-1) of the objects linking the connection points: G α C^(3υ + 1)/(3υ − 1)^ [26]. We have applied this approximation to pure metallo-organic polymer (Figure 6a) and pure iPS gels (Figure 6b) as well as to the hybrid gels with fixed iPS polymer concentration 4%, g/mL (Figure 6a) and fixed metallo-organic polymer concentration 1.4%, g/mL (Figure 6b) and obtained the fractal dimensions shown in Table 2.

In the case of pure iPS gel, the obtained value is 1.5, which would correspond to polymer fibers swollen by the solvent. If we focus on the fractal dimensions of the hybrid gels, the obtained values (1.4 for hybrid gels with fixed iPS concentration and 1.5 for hybrid gels with fixed metallo-organic polymer concentration) are similar to the fractal dimension of pure iPS gel, and far from the fractal dimension of the pure metallo-organic polymer gel (2.5) [15]. This would indicate that the iPS determines the structure and the viscoelastic properties of the hybrid gel, even though the two networks are formed independently.

**Figure 7:**
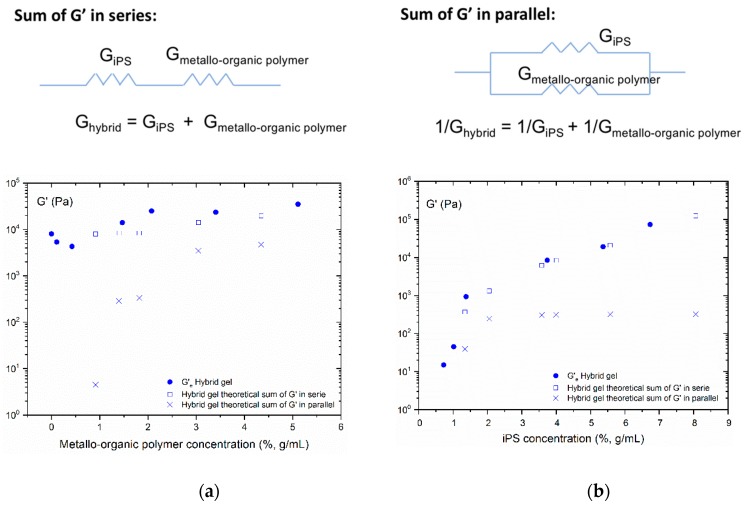
Pictorial scheme of the applied models. Representation of the experimental equilibrium storage modulus as a function of (**a**) metallo-organic polymer concentration (●) and (**b**) iPS concentration (●) and their corresponding theoretical sum of modules in parallel (x) and theoretical sum of the modules in series (□).

When a material is deformed, its elastic behavior can be outlined simply by the Hook’s law. However, if the material is multicomponent, as it is the case for the hybrid gel that is composed of two polymeric gels (iPS and metallo-organic polymer), there is more than one force constant responsible for the final module of the system. Therefore, it is necessary to find a model that could consider these constants so that the resulting force constant describes the elastic behavior of the hybrid gel. Therefore, in order to determine the contribution that each individual gel (iPS gel and metallo-organic polymer gel) has in the storage modulus of the hybrid gel, we have suggested the following two theoretical models. (i) Sum of the individual gel modulus in series: The force constants will act independently against an external force, initially deforming the less rigid spring, and later, the one with the highest rigidity (see the corresponding scheme in Figure 7). (ii) Sum of the modules of the individual gels in parallel: The force constants of the individual gels are interdependent, in such a way that the elastic response of the material is limited by the system whose force constant is higher (see the corresponding scheme in Figure 7).

In Figure 7a,b, experimentally obtained equilibrium storage moduli of the hybrid gel are compared with the values determined through the application the two theoretical models from the experimental data. As concluded from both graphs, the theoretical model that best describes the experimental elastic behavior of the hybrid gel is the one that considers the elastic behavior of the hybrid gel as the sum of individual gel’s storage modulus in series. That is, the hybrid gel would consist of two networks of pure polymers independent of each other.

**Figure 8:**
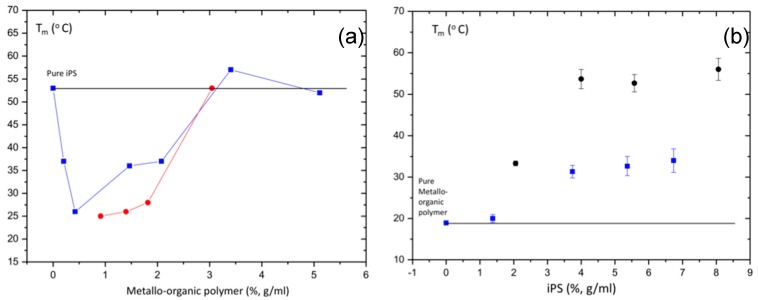
(**a**) Melting temperature (Tm) as a function of metallo-organic polymer concentration (%, g/mL) for pure metallo-organic polymer gel (red circle) and iPS/metallo-organic polymer hybrid gel with a fixed iPS concentration of 4%, g/mL (blue squares). (**b**) Melting temperature (Tm) as a function of iPS concentration (%, g/mL) for pure iPS polymer gel (black circle) and iPS/metallo-organic polymer hybrid gel with a fixed metallo-organic polymer concentration of 1.4%, g/mL (blue squares).

For the sake of comparison, in Figure 8a, we have collected the melting temperatures (Tm) obtained for pure metallo-organic polymer gels and iPS/metallo-organic polymer hybrid gels with a fixed iPS concentration of 4%, g/mL and represented as a function of metallo-organic polymer concentration (%, g/mL). From the figure, we observe how the melting temperature values of the metallo-organic polymer gels increase with the concentration in the studied range of concentrations. In contrast, the melting temperatures of the hybrid gels decreases up to a concentration of metallo-organic polymer of 0.4%, g/mL; beyond this concentration, the melting temperatures increase to values slightly above the pure iPS gel of 4% (g/mL). The melting temperatures of the hybrid gels are lower than those of the pure iPS gel (4%, g/mL) in almost the entire range of metallo-organic polymer concentrations. Therefore, at low metallo-organic polymer concentrations, dilution phenomena may occur, preventing the gelation of the iPS, which causes the melting temperature of the hybrid gel to decrease. This confirms the idea of the formation of the hybrid gels, from the gelation of both iPS and metallo-organic polymers independently, as long as the two polymers are in concentrations above the critical gelation concentrations.

Figure 8b shows the melting temperatures of pure iPS gels and hybrid iPS/metallo-organic polymer gel with a fixed metallo-organic polymer concentration (1.4%, g/mL) as a function of iPS concentration. As observed, melting temperature values of pure iPS gels increase with the concentration of iPS. This increase seems to stabilize at concentrations of 4%, g/mL, above which Tm keeps constant. Similar to what occurs for pure iPS gels, the melting temperatures of the hybrid gels also increase with iPS concentration until it reaches a concentration (close to 4%, g/mL) at which it remains constant. Besides, hybrid gels present melting temperatures higher than the pure metallo-organic polymer gel of 1.4%, (g/mL) (18 °C) but significantly lower than the melting temperature values of the pure iPS gels at each iPS concentration.

When the concentration of iPS in the hybrid gel is not enough for the polymer to undergo gelation on its own, what we are really observing is the melting of the metallo-organic polymer. But, with enough concentration of iPS to gelate on its own, the melting temperature trend observed for the hybrid gels is the same as that of the pure iPS gels. The main difference is that the melting temperature values of hybrid gels are much lower than those of pure iPS gels. It is probably that the observed melting may correspond to the iPS gel, but the temperatures at which this melting occurs are lower due to a dilution effects in the blend.

### 3.3. Morphology of the Hybrid iPS/Metallo-Organic Polymer Gels

**Figure 9:**
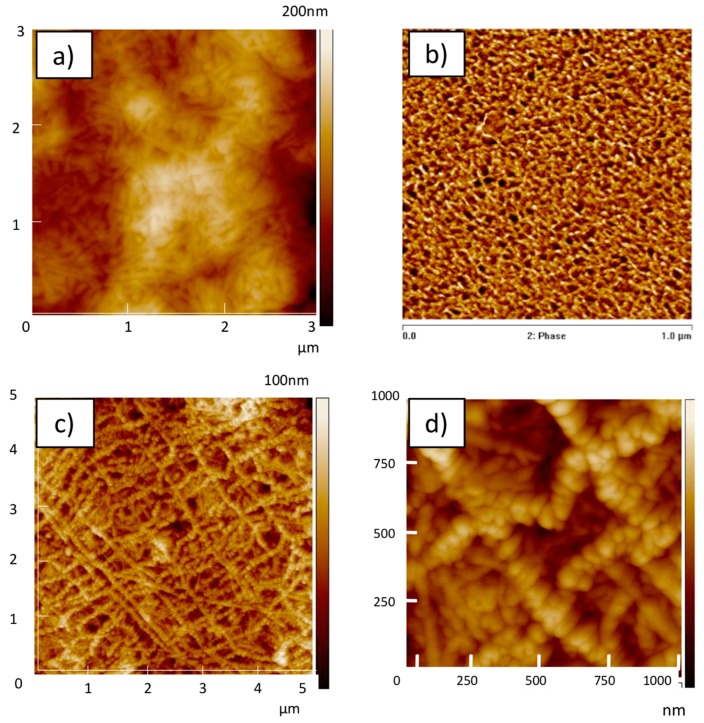
Representative atomic force microscopy (AFM) micrographs corresponding to (**a**) pure iPS xerogel, (**b**) pure metallo-organic polymer xerogel, and (**c**,**d**) hybrid iPS/metallo-organic polymer gel (with iPS/metallo-organic polymer ratio of 97/3).

We performed a morphological study by means of AFM technique and obtained micrographs corresponding to the pure iPS, pure metallo-organic polymer, and hybrid iPS/metallo-organic polymer xerogels, as shown in Figure 9. If we focus on the images corresponding to the pure iPS and pure metallo-organic polymer xerogels, in both micrographs, a network morphology typical of a gel-like structure can be observed. The iPS xerogel appears to consist of fibers with an average diameter of 25 ± 5 nm (Figure 9a). These values are in accordance with those observed in the literature [27]. In the case of metallo-organic polymer xerogel, this is formed by fibers with a mean diameter of 10.3 ± 2.5 nm (Figure 9b).

Regarding micrographs corresponding to hybrid iPS/metallo-organic polymer xerogel (Figure 9c,d), two networks formed with fibers of different sizes are observed. We have measured the diameters of the fibers found on each of the networks and determined that fibers of one network present a diameter of 26 ± 12 nm. This fiber diameter size is similar to the size of iPS xerogel fibers, therefore we could consider that this network could correspond to the one formed by iPS xerogel. The other observed network is formed by fibers with an average diameter of 105 ± 23 nm. Besides, the fibers forming this network seems to be polymer agglomerates, probably agglomerates of the metallo-organic polymer xerogel fibers. Therefore, from the morphological observation, we could consider that the gels of the two polymers grow independently of each other in the hybrid gel.

## 4. Conclusions

By mixing homogeneous solutions of metallo-organic polymer and iPS in cis-decalin separately, stirring at 100 °C, and cooling said solutions to 0 °C, hybrid gels of metallo-organic polymer and iPS in cis-decaline can be prepared. The obtained hybrid gels can be melted above a certain temperature and re-formed upon cooling below a certain temperature (gelling temperature) at a sufficient cooling rate. These are, therefore, thermo-reversible hybrid gels.

From the study of the thermal properties of the hybrid gels carried out by DSC, three transitions have been determined upon heating: The monotectic transition of the iPS gel, the melting of the iPS gel, and the melting of the metallo-organic polymer gel. The latter two transitions are not always clearly observed, since the endotherms are very wide. All seem to indicate that the gels of the two polymers are formed independently, without affecting each other much, as long as the two polymers are in concentrations above the corresponding critical gelation concentrations.

Through the study of the viscoelastic properties of the hybrid gels, an analogy has been observed between the viscoelastic behavior of hybrid gels and pure iPS gels. Both torque sweeps and temperature sweeps have similar shapes for the different systems. Specifically, in the temperature scans it is possible to observe the two iPS gel transitions seen in the thermal measurements: Monotectic transition and gel melting. Both the equilibrium storage modulus and the melting temperature of the hybrid gels are greater than the equilibrium modulus and the melting temperatures of the gels of the pure polymers when the polymer concentrations are high enough to avoid dilution phenomena in the mixtures. This would indicate the formation of an interpenetrated network of polymer gels, which grow independently.

To determine the structure of the hybrid gels, the approximation for systems with enthalpic elasticity of the De Gennes model was used. By this method, a fractal dimension of 1.4 has been determined for hybrid gels with constant concentration of iPS. This value is similar to that obtained for pure iPS gels and is well below that obtained for pure polymer-metal-organic gels. For hybrid gels with a constant concentration of metallo-organic polymer, the obtained fractal dimension is 1.5, similar to that of the pure iPS gel. In both cases, it can be said that the iPS is the polymer that determines the final structure of the hybrid gel.

Attempts have been made to determine the contributions of the gels of the pure polymers separately to the storage module (G’) of the hybrid gels. It has been observed how the elastic behavior of the hybrid gels fitted best to a model in which the elastic moduli of the gels are added in series. This behavior is typical of systems with two independent phases, in which the iPS governs the elastic behavior.

Through AFM, the presence of two independent networks has been observed in the hybrid xerogel. These networks correspond to the networks of the gels of the two separate polymers, which are growing independently in the hybrid gel forming two phases. These hybrid gels, therefore, are interpenetrated organic/metallo-organic networks.

## Figures and Tables

**Table 1:** Temperatures corresponding to endothermic and exothermic peaks of the pure iPS gel, pure metallo-organic polymer gel, and hybrid gel are collected.

Samples	Endothermic Peaks Temperature (°C)			Exothermic Peak Temperature (°C)
iPS gel	18		43	6
Metallo-organic gel		28		11
Hybrid gel (fixed iPS)	20	30	43	
Hybrid gel (fixed metallo-organic polymer)	16	28	38	

**Table 2:** Fractal dimension obtained by the correlation G α C^(3υ + 1)/(3υ − 1)^ extracted from Figure 6.

Fractal Dimension	Pure iPS Gel	Pure MOP * Gel	Hybrid Gels	
			Fixed iPS Concentration (4%, g/mL)	Fixed MOP * Concentration (1.4 g/mL)
υ^−1^	1.5	2.5	1.4	1.5

* MOP: Metallo-organic polymer.
